# Optimizing Ultrasonic Ellagic Acid Extraction Conditions from Infructescence of *Platycarya strobilacea* Using Response Surface Methodology

**DOI:** 10.3390/molecules15117923

**Published:** 2010-11-04

**Authors:** Liang-Liang Zhang, Man Xu, Yong-Mei Wang, Dong-Mei Wu, Jia-Hong Chen

**Affiliations:** 1 Institute of Chemical Industry of Forest Products, CAF; National Engineering Lab. for Biomass Chemical Utilization; Key and Open Lab. on Forest Chemical Engineering, SFA; Key Lab. of Biomass Energy and Material, Nanjing 210042, China; 2 Institute of New Technology of Forestry, CAF, Beijing 100091, China

**Keywords:** *Platycarya strobilacea*, ellagic acid, ultrasonic extraction, optimization, response surface methodology

## Abstract

The infructescence of *Platycarya strobilacea* is a rich source of ellagic acid (EA) which has shown antioxidant, anticancer and antimutagen properties. Response surface methodology (RSM) was used to optimize the conditions for ultrasonic extraction of EA from infructescence of *P. strobilacea*. A central composite design (CCD) was used for experimental design and analysis of the results to obtain the optimal processing parameters. The content of EA in the extracts was determined by HPLC with UV detection. Three independent variables such as ultrasonic extraction temperature (°C), liquid:solid ratio (mL/g), and ultrasonic extraction time (min) were investigated. The experimental data obtained were fitted to a quadratic equation using multiple regression analysis and also analyzed by appropriate statistical methods. The 3-D response surface and the contour plots derived from the mathematical models were applied to determine the optimal conditions. The optimum ultrasonic extraction conditions were as follows: ultrasonic extraction temperature 70 °C, liquid:solid ratio 22.5, and ultrasonic extraction time 40 min. Under these conditions, the experimental percentage value was 1.961%, which is in close agreement with the value predicted by the model.

## 1. Introduction

Polyphenols are widely distributed in the plant kingdom and are important components of common foods, including tea, red wine, fruits, beverages and various medicinal plants. The importance of polyphenols arises from their effects on sensory properties, including astringency and colour, and possible health effects that they may have. One of the polyphenols is ellagic acid (EA), a dimeric derivative of gallic acid, which mainly exists in higher plants, including fruits and flowers, combined with its precursor, hexahydroxydiphenic acid or bound in the form of ellagitannins [1]. EA was studied in the 1960s, mainly for its effects on blood clotting, its hemostatic activity and its effects in whitening of the skin, but reports about effects of EA on carcinogenesis were published in the decades that followed. Interest in EA has increased during the past few years due to its possible antimutagentic, antiviral and anticarcinogenic effects, proved by several studies, especially in laboratory animals, while a few works have reported results in humans [2,3,4,5]. Some ellagitannins have also been shown to possess anti-tumor-promoting activity, antibacterial and antiviral properties and host-mediated antitumor effects [6]. EA has also shown antioxidant activity as an inhibitor of *in vitro* lipid peroxidation and, because of its combined actions, it is used in the food industry. Extracts from red raspberry leaves or seeds, pomegranates, or other sources are said to contain high levels of EA, and are available as dietary supplements in capsule, powder, or liquid forms. A recent profusion of pomegranate nutraceutical products, ‘‘standardized to 40% EA” has appeared in the marketplace [7]. EA was reported to occur in significant quantities in 46 fruits, including raspberries, strawberries and cranberries, and also in nuts, walnuts, pecans, pomegranates and other plant foodstuffs [1]. Plants produce EA to protect themselves from microbial infection and pests. The barks of trees are rich in polyphenol components which help to protect the trees against predators and pathogens [8]. In the case of *Platycarya strobilacea* Sieb. et Zucc, EA is present in the heartwood, as reported by Tanaka *et al.* [9], but there has not been any study of the concentration of EA in the parts of the infructescence of *P. strobilacea*. 

The efficiency of the extraction of EA from the infructescence of *P. strobilacea* can be affected by many factors including ultrasonic extraction temperature, liquid:solid ratio, and ultrasonic extraction time. In such situations, where multiple variables may influence the extraction yield, response surface methodology (RSM) is an effective technique for optimizing the process [10,11]. The methodology involves three steps: (1) experimental design in which the independent variables and their experimental levels are set using well-established statistical experimental designs such as the central composite design (CCD); (2) response surface modeling through regression analysis; and (3) process optimization using the response surface models. The principles and applications of RSM have been well described [12]. As a powerful statistical and mathematical tool, RSM has a major advantage over the one-factor-a time approach in that it allows the evaluation of the effect of multiple variables and their interactions on the output variables with reduced number of trials [13].

The purpose of the present study was to optimize the process for production of EA from the infructescence of *P. strobilacea* using response surface methodology (RSM) employing a CCD (three factors and five levels) to study the effects of ultrasonic extraction temperature, liquid:solid ratio, and ultrasonic extraction time on the extraction yield of EA.

## 2. Results and Discussion

### 2.1. Statistical Analysis and Model Fitting

**Figure 1:**
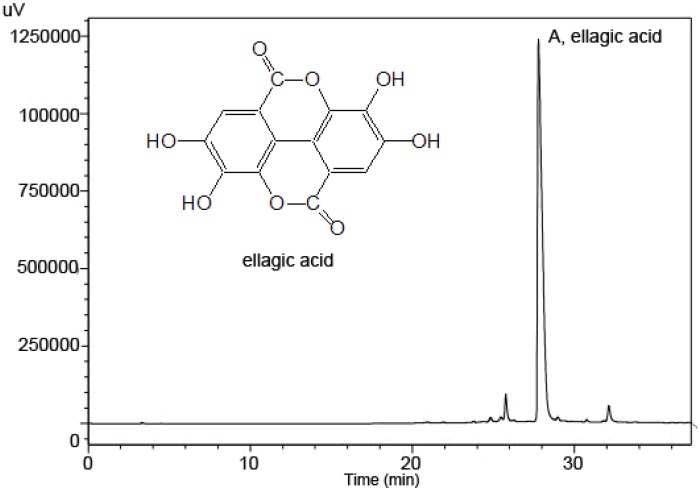
A typical HPLC chromatogram of a methanol extract of the infructescence of *P. strobilacea*, A, ellagic acid.

EA is a major product in the methanolic extracts of the infructescence of *P. strobilacea*, as shown by a typical HPLC chromatogram of the extracts (Figure 1) with detection at 357 nm, as confirmed by the corresponding UV spectrum. 

Response surface optimization is more advantageous than the traditional single parameter optimization in that it saves time, space and raw materials. A total of 20 runs were needed for optimizing the three individual parameters in the current CCD. Table 1 shows the experimental conditions and the EA extraction yield results according to the factorial design. A maximum EA extraction yield of (2.046%) was recorded under the experimental conditions of ultrasonic extraction temperature 70 °C, liquid:solid ratio 25, and ultrasonic extraction time 40 min. By applying multiple regression analysis on the experimental data, the response variable and the test variables were related by the following quadratic equation:

*Y* = 1.61 + 0.27*x*_1_ + 0.095*x*_2_ + 0.28*x*_3_ + 0.073*x*_1_*x*_2_ + 0.061*x*_1_*x*_3_ − 0.065*x*_2_*x*_3_ − 0.31*x*_1_^2^ − 0.084*x*_2_^2^ + 0.033*x*_3_^2^

where *x*_1_, liquid:solid ratio (mL/g); *x*_2_, ultrasonic extraction time (min); *x*_3_, ultrasonic extraction temperature (°C). ANOVA was used to evaluate the significance of the coefficients of the models. The regression coefficient values of equation are listed in Table 2. The *P*-values were used as a tool to check the significance of each coefficient, which in turn may indicate the pattern of the interactions between the variables. For any of the terms in the model, a large regression coefficient and a small *P*-value would indicate a more significant effect on the respective response variables [14]. Thus, the smaller was the values of *P*, the more significant was the corresponding coefficient. It can be seen from this table that the linear coefficients (*x*_1_, *x*_2_, *x*_3_) and the quadratic term coefficients (*x*_1_, *x*_3_) were significant, with very small *P*-values (*P* < 0.05). The other term coefficients were not significant (*P* > 0.05). 

**Table 1:** Response surface central composite design and results for ellagic acid extraction yield.

No.	*X*_1_, liquid:solid ratio (mL/g)	*X*_2_, ultrasonic extraction time (min)	*X*_3_, ultrasonic extraction temperature (°C)	*Y*, extraction yield (%)
1	−1(15)	−1(20)	−1(50)	0.476
2	1(25)	−1(20)	−1(50)	0.883
3	−1(15)	1(40)	−1(50)	0.614
4	1(25)	1(40)	−1(50)	1.081
5	−1(15)	−1(20)	1(70)	1.456
6	1(25)	−1(20)	1(70)	1.877
7	−1(15)	1(40)	1(70)	1.104
8	1(25)	1(40)	1(70)	2.046
9	−1.68(11.59)	0(30)	0(60)	0.415
10	1.68(28.41)	0(30)	0(60)	1.252
11	0(20)	−1.68(13.18)	0(60)	1.125
12	0(20)	1.68(46.82)	0(60)	1.803
13	0(20)	0(30)	−1.68(43.18)	0.695
14	0(20)	0(30)	1.68(76.82)	1.896
15	0(20)	0(30)	0(60)	1.635
16	0(20)	0(30)	0(60)	1.599
17	0(20)	0(30)	0(60)	1.580
18	0(20)	0(30)	0(60)	1.590
19	0(20)	0(30)	0(60)	1.612
20	0(20)	0(30)	0(60)	1.625

**Table 2:** Regression coefficients of the predicted quadratic model.

Parameter	Regression coefficient	Standard error	*t* Ratio	*P*-value
Linear				
*x*_1_	0.27	0.034	0.19	0.0000
*x*_2_	0.095	0.034	0.018	0.0200
*x*_3_	0.40	0.034	0.32	0.0000
Quadratic				
*x*_1_^2^	−0.27	0.033	−0.34	0.0000
*x*_2_^2^	−0.046	0.033	−0.12	0.1942
*x*_3_^2^	−0.11	0.033	−0.18	0.0099
Interaction				
*x*_1_ *x*_2_	0.073	0.045	−0.027	0.1359
*x*_1_* x*_3_	0.061	0.045	−0.039	0.2021
*x*_2_ *x*_3_	−0.065	0.045	−0.16	0.1780

To obtain a simple and yet realistic model, the insignificant terms (*P* > 0.05) are eliminated from the model through a “backward elimination” process. The statistical parameters obtained from the ANOVA for the reduced models are given in Table 3. For the reduced models, *P* < 0.05 is obtained, implying that these models are significant. The adequate precision value is a measure of the “signal (response) to noise (deviation) ratio”. A ratio greater than four is desirable [12,15]. In this study, the ratio was found to be 19.949, which indicates an adequate signal and therefore the model is significant for the extraction process. As can be seen in the Table 3, no interaction between factors is statistically significant for the three responses.

**Table 3:** Reduced response models and statistical parameters obtained from ANOVA (after backward elimination).

Response	Reduced response models *^a^*	Adjusted *R*^2^	Model *P* value	% CV	Adequate precision
*Y*	1.57 + 0.27 *x*_1_ + 0.095*x*_2_ + 0.40*x*_3_ – 0.26*x*_1_^2^ – 0.10*x*_3_^2^	0.9137	<0.0001	11.05	19.949

*^a^* Only significant coefficients with *P* < 0.05 are included. Factors are in coded levels.

### 2.2. Optimization of EA Extraction Conditions

**Figure 2:**
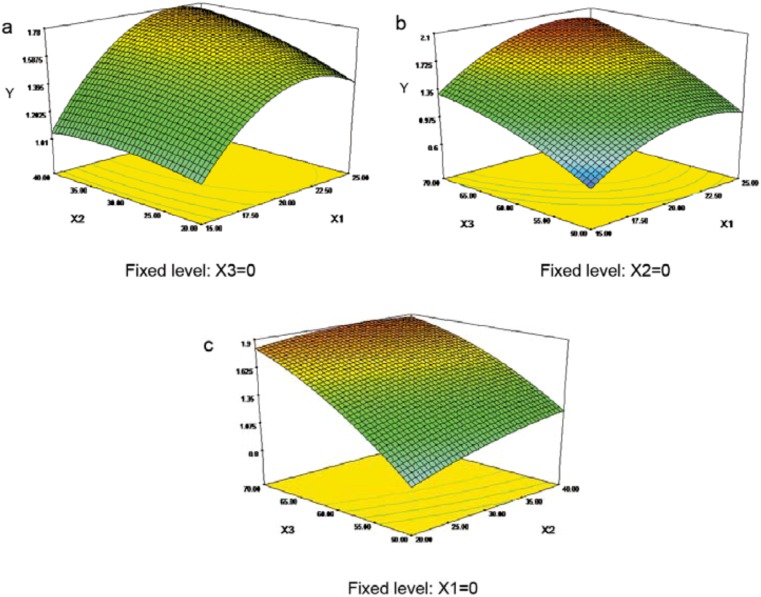
Response surface plots and contour plots showing the effects of variables (*x*_1_: liquid:solid ratio, mL/g; *x*_2_: ultrasonic extraction time, min; *x*_3_: ultrasonic extraction temperature, °C) on the response *Y*.

The graphical representations of the regression equation, called the response surfaces and the contour plots were obtained using Design-Expert software version 7.0, and the results of EA extraction yield as affected by ultrasonic extraction time, liquid:solid ratio, and ultrasonic extraction temperature are presented in Figure 2. 

Response surface methodology plays a key role in identifying the optimum values of the independent variables efficiently, under which dependent variables could achieve a maximum response. In the response surface plot and contour plot, the extraction yield of EA was obtained along with two continuous variables, while the other two variables were held constant at their respective zero level (center value of the testing ranges). In the figures, the maximum predicted value indicated by the surface was confined to the smallest ellipse in the contour diagram. Elliptical contours are obtained when there is a perfect interaction between the independent variables [16,17]. The independent variables and maximum predicted values from the figures corresponded with the optimum values of the dependent variables (responses) obtained by the equations [18,19].

In Figure 2a, when the 3-D response surface plot and the contour plot were developed for the extraction yield of EA with varying ultrasonic extraction time and liquid:solid ratio at fixed ultrasonic extraction temperature (0 level), the extraction yield of EA increased with the increasing ultrasonic extraction time, and increased rapidly with increase of liquid:solid ratio from 11 to 22, then dropped from 22 to 28. The 3-D response surface plot and the contour plot in Figure 2b, which give the extraction yield of EA as a function of ultrasonic extraction temperature and liquid:solid ratio at fixed ultrasonic extraction time (0 level), indicated that the extraction yield of EA was increased rapidly with increase of ultrasonic extraction temperature from 50 to 70 °C. The extraction yield of EA affected by different ultrasonic extraction temperature and ultrasonic extraction time was seen in Figure 2c, when liquid:solid ratio was fixed at zero level. It can be seen that the extraction yield of EA increased with the increasing ultrasonic extraction time and reached the maximum value when extraction time at 40 min.

As shown in Figure 2, it can be concluded that optimal extraction condition of EA from the infructescence of *P. strobilacea* were ultrasonic extraction temperature 70 °C, liquid:solid ratio 22.5, and ultrasonic extraction time 40 min. Among the three extraction parameters studied, ultrasonic extraction temperature was the most significant factor affecting the EA extraction yield, followed by liquid:solid ratio, and ultrasonic extraction time according to the regression coefficients significance of the quadratic model (Table 2) and gradient of slope in the 3-D response surface plot (Figure 2).

### 2.3. Verification of Predictive Model

The suitability of the model equations for predicting optimum response values was tested under the conditions: ultrasonic extraction temperature 70 °C, liquid:solid ratio 22.5, and ultrasonic extraction time 40 min. This set of conditions was determined to be optimum by the RSM optimization approach and was also used to validate experimentally and predict the values of the responses using the model equation. A mean value of 1.961%, obtained from real experiments, demonstrated the validation of the RSM model, indicating that the model was adequate for the extraction process (Table 4).

**Table 4:** Predicted and experimental values of the responses under optimum conditions.

**Optimum conditions**				**EA Extraction yield (%)**	
Liquid:solid ratio	Ultrasonic extraction time (min)	Ultrasonic extraction temperature (°C)		Experimental	Predicted
22.5	40	70		1.961%	2.028%

## 3. Experimental

### 3.1. Materials

Dried infructescence of *P. strobilacea* (moisture 17% in weight) was collected from the ground in Liuan, Anhui Province, China. The infructescence was ground in a knife mill and the powdered sample was sieved to select particles smaller than 1 mm. EA was purchased from Sigma Chemical Co. (St. Louis, MO, USA). The solvents methanol and trifluoroacetic acid (TFA) were of analytical reagent (AR) purity grade. The CH_3_CN used for the analysis were of HPLC grade. Deionized water was used throughout.

### 3.2. Experimental Design

RSM was applied to evaluate the effects of ultrasonic extraction temperature, liquid:solid ratio, and ultrasonic extraction time on the yields of EA. RSM was performed using the Design-Expert software program version 7.0. The coded and uncoded independent variables used in the RSM design are listed in Table 5. The levels of the independent parameters were based on preliminary experimental results. The experimental design was based on the CCD as shown in Table 1. A CCD uses the method of least squares regression to fit the data to a quadratic model. The quadratic model for the response (yield of EA, *Y*) was as follows:

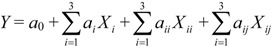

where *Y* represents the response variable, *a*_0_ is a constant, *a_i_*, *a_ii_* and *a_ij_* are the linear, quadratic and interactive coefficients, respectively. *X_i_* and *X_j_* are the levels of the independent variables. The software uses this quadratic model to build the response surface. The adequacy of the model was determined by evaluating the lack of fit, coefficient of determination (*P*-value) and the Fisher test value (*F*-value) obtained from the analysis of variance (ANOVA) that was generated by the software. Statistical significance of the model and model parameters was determined at the 5% probability level (α = 0.05). Three-dimensional surface response plots were generated by varying two variables within the experimental range and holding the other constant at the central point. 

**Table 5:** Uncoded and coded levels of independent variables used in the RSM design.

**Symbols**	**Independent variables**	**Coded levels**				
		−1.68	−1	0	+1	+1.68
*x*_1_	Liquid:solid ratio (mL/g)	11.59	15	20	25	28.41
*x*_2_	Ultrasonic extraction time (min)	13.18	20	30	40	46.82
*x*_3_	Ultrasonic extraction temperature (℃)	43.18	50	60	70	76.82

### 3.3. Extraction

The EA was extracted according to the method described by Bianco *et al.* [8]. An amount of 500 mg (in duplicate) of finally ground sample was suspended in methanol (11.59-28.41 mL) and the mixture was covered to prevent evaporation. The sample was sonicated in a water bath for 13.18-46.82 min at 43.18-76.82 °C. The extract was filtered through Whatman Grade no. 1 filter paper (11 µm) and diluted with methanol to 100 mL. The extracts were analyzed by HPLC.

### 3.4. HPLC-UV Analysis

The polyphenol fraction was isolated by methanolic extraction and EA was determined in the extracts by HPLC with UV detection. The detector was operated at 357 nm wavelength which corresponded to the experimentally found maximum absorption of the EA standard. An aliquot of the extract were filtered through a 0.45 µm syringe filter prior to HPLC-UV analysis. EA was separated using an Inertex C18 column (5 μm, 250 mm × 4.6 mm, Scienhome, China). The solvent flow rate was 1 mL/min and the mobile phase was composed of solvent (A) water (0.1% TFA, v/v) and solvent (B) CH_3_CN with the solvent programmed as follows: 0-8th minutes 7% B, 8th-25th minutes 7%-32% B, 25th-30th minutes 32%-35% B, 30th-35th minutes 35% B. EA peak was identified by comparing its retention time with those of standards and the concentration was calculated from the calibration curves. The analysis was carried out in triplicate.

### 3.5. Statistical Analysis

Experimental data was analyzed by multiple regressions to fit the quadratic equation to all independent variables. Analysis of variance (ANOVA) was performed to evaluate significant differences between independent variables. To visualize the relationships between the responses and the independent variables, surface response and contour plots of the fitted quadratic regression equations were generated using Design-Expert software version 7.0.

## 4. Conclusions

The extraction conditions have a significant effect on the EA extraction yield. The use of the contour and surface plots in RSM was effective for estimating the effect of three independent variables (liquid:solid ratio; ultrasonic extraction temperature in °C; and ultrasonic extraction time in min). The optimum set of the independent variables was obtained graphically in order to obtain the desired levels of EA extraction. An optimal experimental extraction yield of 1.961% was obtained when the optimum conditions of EA extraction (ultrasonic extraction temperature 70 °C, liquid:solid ratio 22.5, and ultrasonic extraction time 40 min) were used. Under these optimized conditions the experimental EA extraction yield agreed closely with the predicted yield of 2.028%.
